# CHRONIC DISEASE AND LIFE EXPECTANCY DISPARITIES OF OLDER ADULTS IN SINGAPORE

**DOI:** 10.1093/geroni/igac059.2575

**Published:** 2022-12-20

**Authors:** Xueying Guo, Hwee lin Wee, Cynthia Chen

**Affiliations:** national university of singapore, Singapore, Singapore; National University of Singapore, Singapore, Singapore; National University of Singapore, Singapore, Singapore

## Abstract

Singapore is one of the countries with longest life expectancy, also one of the most rapidly ageing societies in the world. This study investigates the future demographic transitions in Singapore, focusing on the life expectancy disparities of the older adults. We developed a dynamic Markov microsimulation model based on the Singapore Multi-Ethnic Cohort study. The model was adapted to project future trends of chronic diseases, mental illnesses as well as hospitalisation costs of Singapore older people from 2020 to 2050. The prevalences of diabetes, heart disease, hypertension, stroke, disability, dementia and depression of people aged 51 and above was projected to be increasing. Overall projected life expectancy at age 51 for male is 27.8 years, and for female is 34.5 years. Moreover, for older people with secondary or upper education, the overall projected life expectancy at age 51 is 34.1 years, active life expectancy at age 51 is 28.6 years. For older people with primary or lower education, the overall life expectancy at age 51 is projected to be 27.9 years, and active life expectancy projected to be 23.9 years. Our study has significant social and economic implications for policymakers in the way that it demonstrates disparate trends in aging and costs among different education groups in Singapore.

